# Failure to detect entorhinal grid-like signals in a passive navigation human fMRI study

**DOI:** 10.1162/IMAG.a.1196

**Published:** 2026-04-07

**Authors:** Jonas Kransberg, Anne Cecilie Sjøli Bråthen, Emilie Sogn Falch, Knut E.Ø. Øverbye, Pablo F. Garrido, Anders M. Fjell, Matthias Stangl, Thomas Wolbers, Markus H. Sneve, Kristine B. Walhovd

**Affiliations:** Center for Lifespan Changes in Brain and Cognition, Department of Psychology, University of Oslo, Norway; Department of Physics, University of Oslo, Oslo, Norway; Computational Radiology and Artificial Intelligence, Department of Radiology and Nuclear Medicine, Oslo University Hospital, Oslo, Norway; Department of Biomedical Engineering and Department of Psychological & Brain Sciences, Center for Systems Neuroscience, Cognitive Neuroimaging Center, Neurophotonics Center, Boston University, Boston, MA, United States; Department of Neurosurgery, Boston Medical Center, Boston University Chobanian and Avedisian School of Medicine, Boston, MA, United States; Center for Behavioral Brain Sciences (CBBS), Magdeburg, Germany; Aging, Cognition & Technology Research Group, German Center for Neurodegenerative Diseases (DZNE), Magdeburg, Germany

**Keywords:** aging, entorhinal cortex, grid cells, fMRI, spatial navigation

## Abstract

Grid cells in the human entorhinal cortex (EC) play a critical role in spatial navigation and memory. The EC is also one of the first regions affected by ageing and Alzheimer’s disease. This pre-registered functional magnetic resonance imaging (fMRI) study aimed to detect grid-cell-like signals (GLS) in a passive virtual navigation task. Contrary to our hypotheses and previous findings, we did not observe significant GLS at a population level, even in younger participants. Further exploratory analyses investigated the impact of task-engagement, as inferred from object-location memory performance, and showed no relationship with GLS magnitude. We also examined potential influences of a confounding one-fold directional signal and various data-processing choices but observed no consistent patterns. Our findings, consistent with recent null results from similar studies, suggest that passive navigation paradigms may be insufficient for reliably eliciting grid-like signals in human fMRI.

## 1 Introduction

The entorhinal cortex (EC) plays a crucial role in spatial navigation and memory (Brun et al., 2002; Fyhn et al., 2004; Hafting et al., 2005) and is one of the first regions to show structural decline in the form of cortical thinning in both healthy aging and Alzheimer’s disease (AD; Dickerson et al., 2009; Fjell et al., 2014; Singh et al., 2006). This decline coincides with a progressive loss of navigational abilities in both conditions (Lester et al., 2017). The EC contains grid cells, which are believed to support self-motion-related computations such as self-positioning and path integration (Rowland et al., 2016). Established functional magnetic resonance imaging (fMRI) methods allow for the non-invasive detection of grid-like signals (GLS); macroscopically detected signals thought to represent activity of grid cells (Doeller et al., 2010). FMRI studies using this method have demonstrated reduced GLS in older individuals (Stangl et al., 2018) and in younger individuals with heightened AD risk (Kunz et al., 2015), and findings of GLS have been replicated multiple times (i.e., Constantinescu et al., 2016; Julian et al., 2018; Nau et al., 2018). However, little is known about the plasticity of the EC—specifically whether GLS activity can be modified by behavioral interventions or if these age-related changes can be mitigated.

Neural representations of positional information have been extensively documented in freely moving rodents. These studies have revealed the existence of place cells in the hippocampus (O’Keefe & Dostrovsky, 1971), head direction cells in regions such as the presubiculum and postsubiculum (Taube et al., 1990), and grid cells in the EC (Hafting et al., 2005). Head direction cells fire when the animal’s head faces a particular direction (Taube et al., 1990), while place cells are active at specific spatial locations in an environment (O’Keefe & Dostrovsky, 1971). Grid cells, by contrast, fire at multiple, evenly spaced locations arranged in a hexagonal pattern (Hafting et al., 2005). These grid cells, with varying scales, phases, and orientations, collectively “tile” the environment. This tiling enables precise self-localization and supports spatial computations such as path integration and distance estimation (Rowland et al., 2016).

While evidence of grid cells has been established in rodents (Hafting et al., 2005), bats (Yartsev et al., 2011), and non-human primates (Killian et al., 2012), investigating grid cells in humans is challenging due to methodological constraints. Studies have detected human grid-like activity using invasive electrophysiological single-neuron recordings during neurosurgical procedures (Jacobs et al., 2013; Nadasdy et al., 2017), but such methods are practically limited. The spatial offset between individual grid cells complicates the detection of grid-like activity at the macroscopic level. However, specific movement trajectories can introduce systematic variation in activity indicative of GLS: in adaptation to specific environments, grid cells anchor to external reference frames provided by stationary environmental and sensory cues (Stensola et al., 2015), resulting in a shared angular orientation of grids in both neighboring (Hafting et al., 2005) and distal grid cells (Barry et al., 2007). Such alignment can produce systematic variation in population activity as a function of movement direction, yielding a 60°-periodic (6-fold) modulation detectable with fMRI (Doeller et al., 2010). The precise cellular mechanism underlying this macroscopic signal remains debated. One candidate is directional modulation in conjunctive grid × head-direction cells, whose preferred firing directions are aligned to the grid axes (Sargolini et al., 2006). Another is repetition suppression in grid cells, whereby repeated traversal of grid firing fields along aligned directions produces differential adaptation. Currently, it is unknown which mechanism, or combination thereof, is primarily responsible for the hexadirectional fMRI signal (Bin Khalid et al., 2024).

Building on these properties, Doeller et al. (2010) developed a method for detecting GLS by testing for 6-fold symmetry in BOLD signals as a function of movement direction during virtual navigation. This approach has enabled non-invasive investigations of hexagonal signals in the EC in ageing and AD (Bierbrauer et al., 2020; Kunz et al., 2015; Stangl et al., 2018; See Stangl et al., 2020 for a full overview of the methods and variants of these analyses). Observed declines in GLS align with the EC’s sensitivity to aging and AD-risk. Thus, exploring whether interventions, such as cognitive training, can strengthen or restore these signals presents a potential avenue for mitigating age-related declines in certain aspects of spatial navigation.

The present study is part of the *Set to Change* project, which aims to examine how early-life environmental factors and genetic makeup interact to regulate neurocognitive plasticity across the lifespan. The project recruits monozygotic and dizygotic twins to participate in a 10-week virtual reality navigation training programme. This manuscript reports the project’s pre-registered baseline analyses of the fMRI grid-task, which constitute only one element of a much broader preregistered longitudinal project encompassing behavioral, structural, and molecular measures. The broader preregistration encompasses multiple neuroimaging and behavioral modalities, including planned investigations of training-related change, but the present manuscript reports only baseline fMRI results, that is, an attempt to establish grid-like activity patterns in human EC as a prerequisite for later analyses of training-induced modulation. Following the approach introduced by Doeller et al. (2010), participants were scanned while exposed to a virtual environment to identify signal differences arising from movement alignment versus misalignment with a hexagonal grid pattern.

The present study differs from the approach of Doeller et al. (2010) and most previous investigations of grid-cell-like signals in humans by employing a passive rather than active navigation task. Our decision to use a passive navigation task was informed by encouraging pilot data from collaborating groups, which suggested that such paradigms could elicit grid-like responses. Passive navigation also offered advantages in feasibility, removing barriers posed by controller use that can disproportionately affect older participants. An added benefit was methodological consistency, since identical trajectories for each run ensured that all participants were exposed to the same spatial paths and had consistent sampling of movement directions. However, the effects of passive movement on grid-cell activity are unclear. In rodents, grid cells show robust firing during active self-movement, but passive transport disrupts this activity (Winter et al., 2015). Whether this disruption generalizses to humans is uncertain. Unlike rodents, whose internal state during passive movement can be difficult to assess, we can ensure at least some level of task-engagement in humans. In the present study, an object-location memory task confirmed that participants remained at least somewhat engaged with the task during passive movement.

As outlined in our pre-registration, this study firstly aimed to detect 6-fold symmetrical grid-like activity in the EC at baseline, with the expectation that it would be stronger in younger participants compared to older, consistent with Stangl et al. (2018). The sample included 110 participants (72 female, 38 male; age range 18.2–78.6; mean age 36.5; see Table 1 for sample demographics) who all underwent two runs of a GLS fMRI paradigm. We further hypothesized that control analyses investigating 5-fold and 7-fold symmetry would yield no significant results, and that cases of 6-fold activity would exhibit temporal and spatial stability in grid orientations across EC voxels.

**Table 1.** Descriptives for manually segmented sample.

Age group	n	Age^a^	Sex F/M	IQ^a^	Education	MMSE score^a^	Behavioural task score^a^
Younger	80	27.4 (5.5), 18.2–39.4	56/24	107.3 (11.8), 70–128	15.6 (2.5), 10–21	28.9 (1.3), 26–30	6.4 (0.9), 2.5–7
Older	30	60.8 (11.4), 40.4–78.6	16/14	116.9 (10.3), 100–144	16.6 (2), 12–21	28.7 (1.1), 26–30	5.3 (1.2), 2–7

Age and Education are given in years.

a Values are presented as Mean (SD), Range.

IQ = Intelligence Quotient, MMSE = Mini-Mental State Examination.

Contrary to our expectations, we did not detect consistent grid-like activity in the EC. This aligns with a recent study by Newton et al. (2024), which also failed to detect GLS using an identical passive navigation paradigm. While null findings are rarely emphasized in the grid code literature, they offer an important opportunity to critically evaluate both methodological and analytical approaches. To this end, we present the null findings of our pre-registered analyses and conduct exploratory analyses to systematically investigate potential limitations and alternative explanations.

## 2 Methods

### 2.1 Participants

A total of 227 participants took part in the *Set to Change* twin project at the Centre for Lifespan Changes in Brain and Cognition (LCBC), Department of Psychology, University of Oslo. Of these, 219 underwent MRI scanning. Twelve participants were excluded due to missing or unusable data: eight did not complete one or both fMRI runs, one dataset was lost due to human error, one was excluded due to radiological findings, and two were excluded due to severe MRI artifacts. EC masks were manually delineated for a subset of participants yielding a final sample of 110 participants; see Table 1 for sample demographics. For exploratory analyses using automatic EC segmentation, data for the whole available fMRI sample of 207 participants were used; see Table 2 for full fMRI sample demographics.

**Table 2.** Descriptives for full fMRI sample.

Age group	n	Age^a^	Sex F/M	IQ^a^	Education	MMSE score^a^	Behavioural task score^a^
Younger	137	26.0 (5.8), 16.2–39.7	107/30	105.8 (11.9), 63–132	15.5 (2.5), 10–21	28.7 (1.3), 23–30	6.4 (0.9), 2.5–7
Older	70	57.2 (11.8), 40.4–78.6	34/36	115.5 (10.3), 80–144	16.3 (2.6), 10–22	28.8 (1.1), 25–30	5.3 (1.4), 1.5–7

Age and Education are given in years.

a Values are presented as Mean (SD), Range.

IQ = Intelligence Quotient, MMSE = Mini-Mental State Examination.

Although this study does not include twin-specific analyses, all participants were same-sex twins recruited as community volunteers, with the majority drawn from the Norwegian Twin Registry (NTR; Nilsen et al., 2019) and the remainder via online advertisements. All procedures were approved by the regional Ethical Committee of Southern Norway, and all participants provided written informed consent.

Each participant underwent a health screening and met the following inclusion criteria: age 16 years or older, fluent Norwegian speaker. Participants were recruited as healthy volunteers from the community. While we prioritized individuals without cognitive concerns or neurological conditions, inclusive recruitment criteria were adopted to maximize twin-pair participation. Younger participants were not excluded based on cognitive functioning, but participants aged 40 and older were required to score at least 25 on the Mini-Mental State Examination (MMSE; Folstein et al., 1975). For age comparisons, participants were categorizsed into younger and older groups based on a cutoff age of 40 years, with those younger than 40 assigned to the younger group and those 40 or older to the older group.

### 2.2 Grid code task

**Fig. 1.**
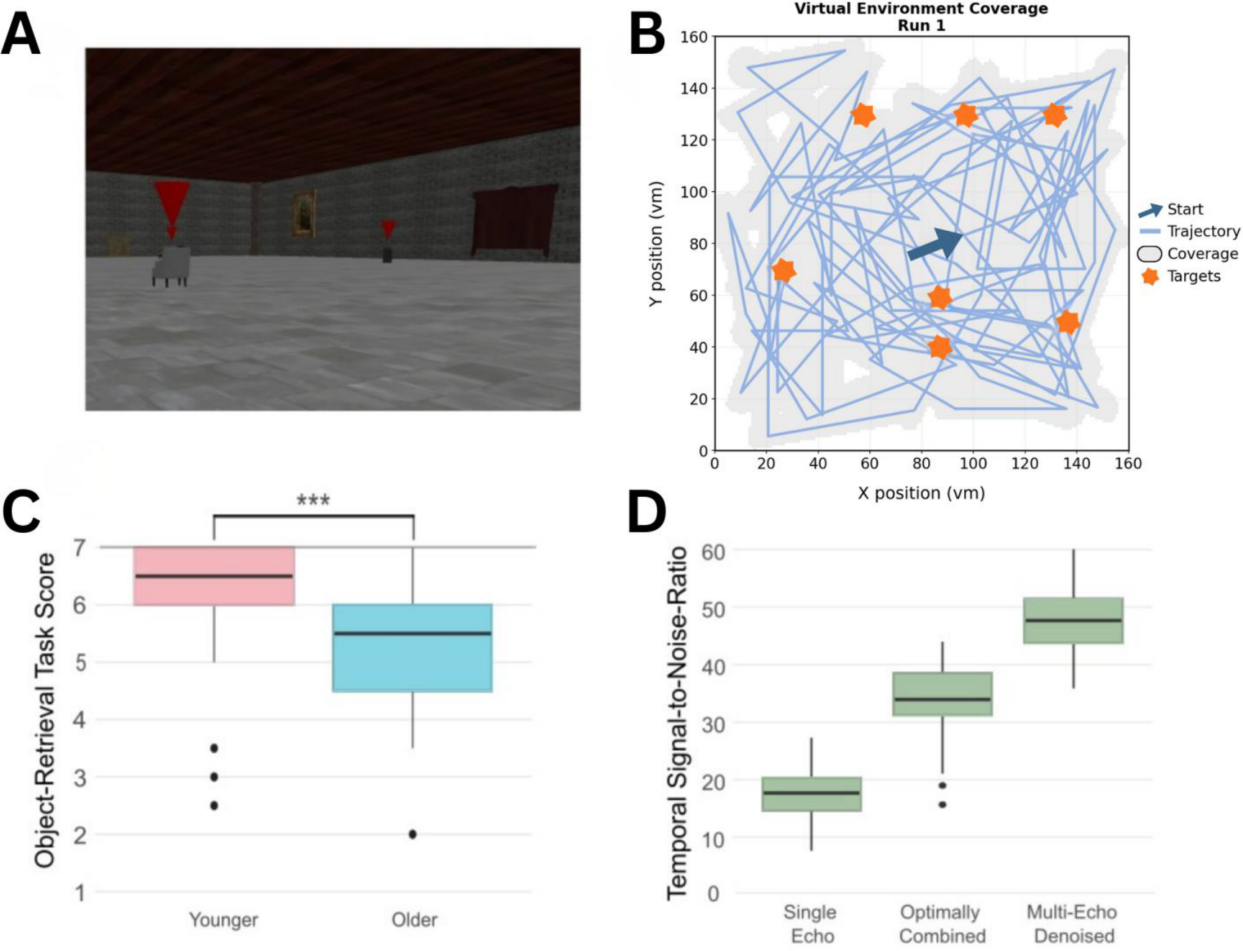
fMRI behavioural task and data acquisition. (A) Participants performed an object-location memory task where they passively moved around a virtual environment. Participants were asked to remember the locations of seven red cone marked objects. Following each run, participants had to indicate correct placements for each of the target objects. (B) Plot showing participant trajectory in the virtual environment for run 1. Blue arrow indicates starting point, pink stars indicate target locations, and the green line is the trajectory through the environment. The grey area indicates the covered area through the trial. See methods for calculation of environment coverage. (C) Boxplot of behavioral task scores averaged between runs for the two age groups. There were noticeable ceiling effects in both groups and a significant difference between the groups. ***p < 0.001. (D) Temporal signal-to-noise ratio (tSNR) calculated from EC voxels at different fMRI data processing stages. Single Echo indicates tSNR from just one echo (TE = 30.9 ms), Optimally Combined (TEs = 12.2 ms, 30.9 ms, 49.6 ms) is the combined images of three echoes, and Multi-Echo Denoised indicates tSNR from the final optimally combined image after multi-echo ICA-based denoising.

Each participant performed three runs of a passive movement object-location memory task in a virtual environment (Fig. 1A). The first run served as a training session conducted on a laptop before MRI scanning, while runs two and three were performed inside the scanner. Each run lasted 620 seconds which yielded 1240 seconds (i.e., >20 minutes) of actual in-scanner task time. Participants were instructed to remember the locations of seven target objects placed in the virtual environment. These objects were each marked with a hovering red cone.

To ensure experimental control, movement trajectories were generated randomly once per run and then fixed, such that all participants experienced the same paths within each run. Participants have no control over navigation; they were instructed to mentally immerse themselves in the environment, focus on object locations, and remain attentive throughout the task. The movement trajectory consisted of separate translational and rotational movements, but never both simultaneously. The virtual environment was a 160 x 160 virtual meter (vm) room, with movement speed set to 15 vm/s and rotation speed at 50°/s. The virtual camera height was set to 1.7 vm. Movement directions were sampled across the full 360° range, categorized into 10° bins. Sampling was balanced across directions, with a total of 112 movement segments, yielding 3–4 trials per directional bin within each fMRI run. Participants shared movement trajectories for each run, with each run having its own trajectory. For run 1 participants traveled a total of 5287.85 vm, with an average segment length of 52.50 vm and average time moving in each segment was 3.54 seconds. Rotational movements averaged 102.36°, with a minimum of 10° and a maximum of 170°. Run 2 yielded a total path length of 5698.54 vm, with an average segment length of 50.88 vm and an average segment duration of 3.39 seconds. Average rotational movements were 105.68°, again ranging from 10° to 170°. The percent of the virtual environment covered in each run was calculated by discretising the environment into 1 x 1 vm spatial bins and marking a bin as covered whenever it fell within the defined coverage geometry, which included a general radius around the participant of 3 vm and a cone projecting in the direction of movement 8 vm ahead with a 65 degree width. This yielded a 75.19% coverage of the virtual environment in the first fMRI run, and 75.65% coverage in the second fMRI run.

Each run was followed by a memory test. Participants were presented with three images per object—one showing the correct placement and two distractor locations. They were asked to verbally indicate the correct placement. The average object placement scores across the two runs within the scanner were used for subsequent analyses. The task was implemented in Unity (version 2018.3.6f1) and presented via an MRI-compatible screen (NordicNeurolab, Bergen, Norway) viewed through a mirror mounted on a head coil.

### 2.3 Imaging procedures

MRI data were collected on a Siemens Prisma 3T with a 32-channel head coil. Anatomical scans were recorded using a T1-weighted MPRAGE with the following parameters: repetition time (TR) = 2400 ms, inversion time (TI) = 1000 ms, echo time (TE) = 2.22 ms. In addition, T2-weighted SPACE anatomical scans were obtained with the following parameters: TR = 3200 ms, TE = 563 ms. A high in-plane resolution T2-weighted turbo spin echo (TSE) acquisition (0.4 x 0.4 x 2.0 mm), with slices perpendicular to the hippocampal longitudinal axis was acquired to facilitate delineation of the ECs.

Functional MRI was performed using a multi-echo multiband gradient recalled echo (GRE) planar imaging (EPI) sequence with the following parameters: TR = 1000 ms, TEs = [12.2 ms, 30.9 ms, 49.6 ms], MB-factor = 4, flip angle = 63 degrees, GRAPPA factor = 2, partial Fourier = 7/8, matrix size = 84 x 84, FOV = 218 x 218 mm, and slice thickness = 2.6 mm, resulting in an in-plane resolution of 2.6 x 2.6, number of slices = 52, interleaved slice acquisition. All datasets underwent minimal preprocessing using fMRIPrep (Esteban et al., 2019). To mitigate head motion artifacts, we applied a 24-parameter denoising strategy (Ciric et al., 2017). Furthermore, susceptibility-induced distortions, particularly relevant in the EC, were corrected using spin-echo field maps (AP/PA phase-encoding) acquired immediately prior to functional runs. Multi-echo specific processing included optimal combination of individual echoes (Posse et al., 1999) and denoising using *Tedana* (DuPre et al., 2021). *Tedana* employed Principal Component Analysis (PCA) using a moving average (stationary Gaussian) process (Li et al., 2007) to reduce dimensionality. Independent Component Analysis (ICA) was then used to decompose the reduced dataset. Component selection followed the Kundu decision tree (v2.5; Kundu et al., 2013) to classify BOLD (TE-dependent), non-BOLD (TE-independent), and low-variance components. A minimum image regression approach was applied to remove spatially diffuse noise.

Unsmoothed data were used for the majority of the analyses, but for the analyses of smoothed data 4 mm full-width at half-maximum (FWHM) Gaussian kernel smoothing was used. Primary analyses were conducted using manually segmented EC masks (n = 110), whereas exploratory analyses included automatic segmentations to access a larger sample (n = 207).

To assess signal quality across preprocessing pipelines, we computed the temporal signal-to-noise ratio (tSNR) for each voxel within the EC. tSNR was calculated as the mean of each voxel’s time series activity divided by its standard deviation. Voxels with low tSNR were excluded if their values fell more than 1.5 interquartile ranges below the first quartile of the tSNR distribution across voxels within each subject.

### 2.4 Entorhinal cortex region of interest masks

Manually segmented EC ROIs were delineated using ITK-Snap (software version 3.6.; www.itksnap.org; Yushkevich et al., 2016) on participants’ T1-weighted and T2-weighted images following the segmentation protocol provided by Berron et al. (2017). To ensure the reliability of manual segmentations, a second rater independently inspected all EC masks for quality control and accuracy. Any discrepancies were discussed and resolved by consensus. These manually segmented ROIs served as the basis for all primary analyses. Due to the time-consuming nature of manual segmentations, they were only performed for a subset of our participants. To assess effects of segmentation type, and take advantage of the full sample, we ran additional control analyses using automatically segmented masks. The automatic EC ROIs were defined for all participants using FreeSurfer (version 7.3.2) based on cortical parcellations from the Desikan-Killiany atlas. Both manual and automatic segmentations were registered to the pre-processed BOLD data. The resulting probability maps were thresholded at 0.5 such that voxels with a probability of representing the EC greater than or equal to 0.5 were included in the final binary mask. It should be noted that automatically derived EC masks may include portions of adjacent medial temporal lobe cortex, including parts of the perirhinal cortex, whereas manual segmentations are typically more conservative in anatomical extent (see Supplementary Fig. S1).

### 2.5 Analyses of grid-cell like representations

Grid cell-like representations in the EC were analyzed with a Python-adapted version of the code from the Grid Code Analysis Toolbox (GridCat) developed by Stangl et al. (2017), and with a similar approach to that described by Doeller et al. (2010). First, we split each fMRI run into two halves: the first half served as the estimation dataset, and the second half served as the test dataset. We then modeled voxel-wise grid-orientations by fitting the estimation data with a first general linear model (GLM1). This model included two parametric modulators for all translational movement events, specifically sine and cosine of the movement angle multiplied by six (sin(6ϕ) and cos(6ϕ)). Here, (ϕ) represents the angle of movement relative to a fixed reference point in the virtual environment.

Beta values associated with both parametric regressors were extracted for each voxel. We calculated each voxel’s grid orientation via arctan [(β_1_)/(β_2_)]/6 and its signal amplitude via √(β_1_² + β_2_²). We then computed the mean grid orientation (ϕ̄) across voxels using amplitude-weighted circular averaging, where each voxel’s orientation was weighted by its signal amplitude. Next, we fitted the test dataset using a second general linear model (GLM2), which included a parametric modulator reflecting how each event’s movement angle deviates from the mean of all the grid orientations across voxels (ϕ). Specifically, we used cos[6*(ϕ – ϕ̄)], which quantifies how well the observed orientations in the test data align with the estimated grid orientation from the estimation data. The resulting parameter estimates in the EC provide a measure of the magnitude of grid-cell-like representations. When orientations remain stable between the two halves, these parameter estimates are expected to be positive. All steps of this procedure were performed independently for the left and right EC masks, yielding hemisphere-specific grid orientations and GLS magnitudes. This analysis procedure was performed separately for each hemisphere as well as for each of the two fMRI runs. The resulting GLS magnitude estimates were then averaged across the two runs for each participant before being used in subsequent statistical analyses.

To assess the specificity of 6-fold GLS, we conducted additional control analyses for alternative symmetries using the same grid-code analysis framework. In addition to the 6-fold model, we generated models for 5-fold, 7-fold, and 1-fold symmetries. For each symmetry model, we applied the same two-stage GLM approach used for the 6-fold signal, using the desired rotational symmetry instead of six. This approach allowed us to assess whether observed 6-fold symmetry was specific to grid-cell-like activity or whether non-canonical patterns could explain the observed results. One-fold signals, in particular, served as a proxy for head direction signals, as these represent unidirectional modulation of voxel activity rather than the multi-directional firing patterns expected from grid cells.

We validated our Python implementation against GridCAT on each of the two fMRI runs. Using the manually segmented participant subset, Pearson correlations of each participant’s mean GLS closely matched GridCAT (Run 1: r = 0.971; Run 2: r = 0.990; overall: r = 0.980; n = 110).

### 2.6 Analyses of representational stability

Both temporal and spatial stability can affect the ability to detect grid-cell-like representations. Spatial stability refers to the degree to which voxels within an ROI exhibit a consistent non-uniform distribution of grid orientations. When the preferred grid orientations of voxels within the EC are randomly distributed, the resultant mean orientation becomes arbitrary, leading to poor alignment when modelling translation events relative to that mean. Rayleigh’s test for non-uniformity of circular data can be used to test whether the grid orientations of different voxels are uniformly distributed or if the voxels tend towards sharing common orientations (Kunz et al., 2015; Stangl et al., 2017). A higher Rayleigh’s Z then indicates higher spatial stability.

Even if an ROI’s voxel orientations are spatially stable, changes in orientation over different data halves can disrupt the detection of grid-cell-like representations. Temporal stability is assessed by comparing each voxel’s estimated orientation in one half of the data with its orientation in the other half. Voxels whose orientations remain within +-15 degrees are considered temporally stable, and a higher proportion of such voxels within an ROI indicates more robust temporal stability (Kunz et al., 2015; Stangl et al., 2017). High temporal stability suggests that the observed grid orientations are not merely transient fluctuations, but rather reflect a persistent, directionally modulated neural signature.

### 2.7 Statistical analyses

To assess whether GLS magnitudes significantly differed from zero, we conducted one-sample t-tests on the 6-fold, 5-fold, and 7-fold models. For multiple comparison correction, we treated the primary 6-fold hypothesis (2 tests: left and right EC) and control analyses (4 tests: 5-fold and 7-fold in each hemisphere) as separate families. Bonferroni-corrected thresholds were α = 0.025 for primary analyses and α = 0.0125 for control analyses. These tests were also applied separately for the younger and older groups and for participants with high performance on the behavioral task (i.e., those scoring ≥6 out of a maximum of 7). A one-sample t-test was also conducted to compare temporal stability against the chance level of 50%. Welch’s t-tests, accounting for unequal sample sizes, were used to compare GLS magnitudes, temporal stability, and spatial stability between age groups. All analyses were conducted with a significance threshold of p < 0.05. To distinguish between data insensitivity and genuine evidence for the null hypothesis, we supplemented all one-sample and independent-samples t-tests with Bayesian equivalents (Cauchy prior, r = 0.707). Bayes factors are reported as bf_01_ (evidence for null) or bf_10_ (evidence for alternative), with values > 3 considered moderate evidence.

In addition to group comparisons using t-tests, we employed linear mixed-effects models (LMMs) using the lmer() function from the lmerTest package in R (Kuznetsova et al., 2017) to assess continuous relationships between grid-code signals and participant variables. These models allowed us to account for repeated measurements by including subject-specific random intercepts and were estimated using restricted maximum likelihood (REML). The model was specified as follows:

$$\begin{array}{l} 6\text{-fold Signal Magnitude}∼\text{Age}\;\;\times \;\;\text{EC Mask Hemisphere}\;\\ \;+\;\text{Sex}\;+\;(1|\text{Participant}) \end{array}$$

This model examined whether grid-code signals varied as a function of continuous age, hemisphere, sex, and their interactions. We applied the same model when investigating the presence of unimodal signals as well, using grid code betas based on 1-fold rotational symmetries, as opposed to 6-fold. Additionally, to test whether the 1-fold signal influenced the other symmetries we ran two separate LMMs using the following model specification:

$$\begin{array}{l} \left(7\;\text{or}\;6\text{-fold}\right)\text{ Signal magnitude}∼1\text{-fold signal}\;\\ \;\times \;\text{EC Mask Hemisphere}\;+\;\text{Age}\;+\;(1|\text{Participant}) \end{array}$$

The first model assessed whether the 1-fold signal predicted changes in the 7-fold signal, while the second model tested whether the 1-fold predicted changes in the 6-fold signal. Both models included random intercepts to account for repeated measures and were also estimated using REML.

Comparisons between smoothed and unsmoothed GLS estimates, GLS measures from manual and automatic segmentations, and task performance against both 6-fold and 1-fold signals were all conducted using Pearson correlations.

## 3 Results

### 3.1 Primary analyses

We first evaluated whether GLS in the ECs significantly differed from zero under a 6-fold symmetry model. Neither of the two EC masks demonstrated significant grid-like signals (right EC: t(109) = 0.728, p = 0.234, bf_01_ = 7.31, d = 0.07; left EC: t(109) = −0.443, p = 0.671, bf_01_ = 8.60, d = −0.04).

**Fig. 2.**
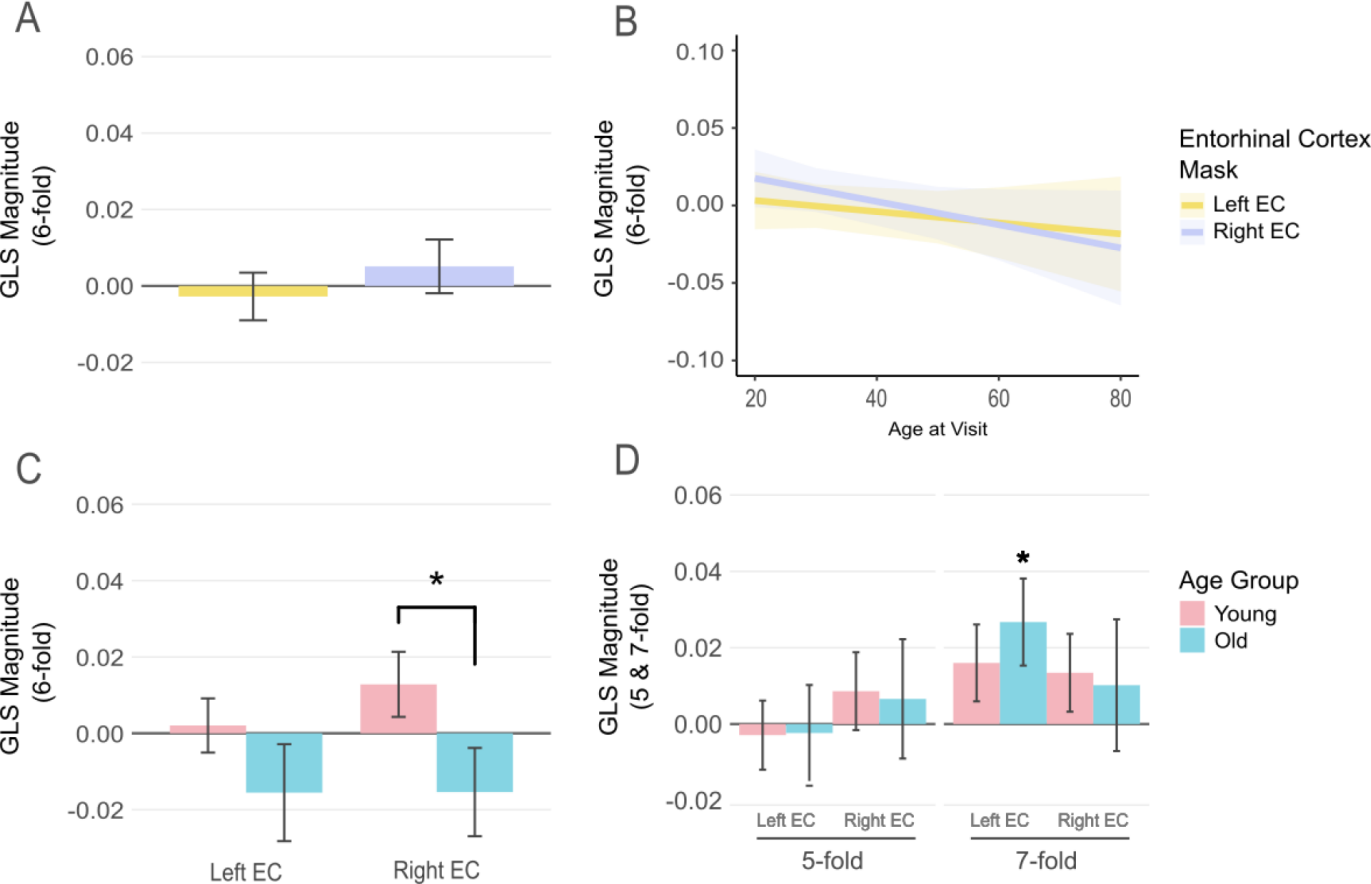
Grid-Like Representations by Age, Hemisphere, and Rotational Symmetry. (A) Mean grid-like signal (GLS) magnitude within the left and right ECs across all participants. No hemisphere showed activity significantly different from zero. (B) Linear mixed-effects model (LMM) analysis of GLS with 6-fold symmetry, showing no significant relationship with age or age x hemisphere interaction, while controlling for sex. (C) GLS split by age group and hemisphere. While no significant one-sample differences were found within groups, there was a significant difference in GLS between younger and older participants in the right EC. (D) Control analyses for 5-fold and 7-fold rotational symmetries. No significant age-group differences were observed for either fold symmetry or EC mask. However, a significant 7-fold signal was detected in the left EC of older participants in a one-sample test. Error bars represent SEM. *p < 0.05.

In our control analyses using 5-fold and 7-fold symmetrical models, conditions under which we did not expect significant GLS, we did, however, observe one significant result. While most 5-fold and 7-fold analyses yielded non-significant results (p > 0.05), a statistically significant signal emerged in the left EC when using the 7-fold model (t(109) = 2.383, p = 0.009, bf_10_ = 1.57, d = 0.23, see Fig. 2). This unexpected finding did not correspond to our hypotheses or previous literature. Prior studies have typically focused on younger samples (e.g., ages 18–31; Bellmund et al., 2016; Doeller et al., 2010) and have found diminished GLS in older participants (Stangl et al., 2018). Given our sample range of 18.2–78.6 years (see Table 1), we divided participants into two age groups (<40 and ≥40 years) to maximize sample retention.

For younger participants neither the right EC (t(79) = 1.5, p = 0.068, bf_01_ = 2.76, d = 0.17) nor left EC (t(79) = 0.28, p = 0.387, bf_01_ = 7.80, d = 0.03) showed significant GLS effects. Control analyses of alternative symmetries yielded similar non-significant results. In the older group, we again did not detect significant 6-fold signals; however, significant 7-fold signals were once again observed in the left EC. In the right EC, a two-sample Welch test comparing the younger (<40) and older (≥40) groups revealed significantly greater grid-code signals in the younger group, compared to the older (t(62.29) = 1.964, p = 0.027, bf_10_ = 1.76, d = 0.042), in alignment with our hypotheses. However, this effect did not survive controlling for multiple comparisons. The group difference was not present in the left EC. There were also no detectable group differences in any of the 7-fold or 5-fold control analyses. Although absolute GLS magnitudes did not significantly differ from zero in either age group, relative comparisons still showed age-related differences. This suggests that although GLS expression was weak across the sample, younger individuals still exhibited relatively higher magnitudes than older adults, consistent with prior findings (Stangl et al., 2018).

We performed a linear mixed-effects analysis to examine whether grid-code signals vary continuously with age while accounting for hemisphere and sex. In this model, the dependent variable was the grid-code signal, with fixed effects for age, hemisphere (comparing the right EC to the left EC reference), sex, and their interaction (age × hemisphere). A random intercept for subject was included to account for repeated measurements. The model was estimated using REML on 220 observations from 110 participants. The mean-centered intercept, representing the estimated signal for a female participant in the left EC at age 36.49, was not significantly different from zero (β = -0.0024, p = 0.755).

The main effect of age was negative (β = –0.00036, SE = 0.00040, t(204.02) = –0.891, p = 0.374), suggesting a trend in which grid-code signals would decrease with increasing age, but this effect did not reach significance. Likewise, the main effect of ROI for the right EC was non-significant (β = 0.0079, SE = 0.0086, t(108) = 0.919, p = 0.360), and there was no significant effect of sex (β = –0.001079, SE = 0.01079, t(108) = –0.099, p = 0.922). Additionally, the interaction between age and the right EC failed to reach significance (β = –0.00039, SE = 0.00051, t(108) = –0.757, p = 0.451).

We supplemented our pre-registered analysis with Bayesian t-tests (Cauchy prior, r = 0.707). The analyses revealed moderate evidence in favour of the null hypothesis in the left EC for the younger age group (bf_01_ = 7.8), while evidence was inconclusive for the older age group (bf_01_ = 2.60). In the right EC the evidence was also in favour of the null, but weaker (bf_01_: Younger = 2.76, Older = 2.31), suggesting that the data in this region were insensitive rather than definitive evidence of absence. While comparisons between the age groups revealed significantly greater GLSs in the right EC of the younger group compared to the older group, the corresponding Bayesian independent-samples t-tests yielded a Bayes Factor of bf_10_ = 1.76, indicating that the evidence for a group difference is inconclusive. Thus, while the direction of the effect aligns with prior literature, the statistical evidence for a robust age difference in the present study is weak.

Bayesian t-tests for the 5-fold control analyses for both hemispheres and age groups yielded moderate evidence for the null hypothesis (bf_01_ > 4.7). The 7-fold model analyses yielded moderate evidence for the null hypothesis in the younger right EC (bf_01_ = 3.54) and the older right EC (bf_01_ = 4.38), while evidence was inconclusive in the younger left EC (bf_01_ = 2.44). Although the initial t-tests showed a significant 7-fold modulation in the left EC of the older participants (p = 0.009), Bayesian analysis failed to provide substantial support for the alternative hypothesis (bf_10_ = 2.02), indicating that the data are not sufficiently informative to confirm the presence of a true effect despite the significant p-value.

Overall, these results indicate that grid-code signals, as measured in our sample, do not vary systematically with age, hemisphere, or sex. While the initial analyses suggested an expected potential age-group difference in GLS and an unexpected 7-fold modulation in older adults, Bayesian analyses characterised both findings as inconclusive. None of our pre-registered hypotheses were robustly supported, and observed deviations from the null hypothesis likely reflect data insensitivity rather than stable neural signals.

### 3.2 Temporal and spatial stability analyses

The absence of stable GLS effects could stem from insufficient temporal (within-subject) or spatial (across-voxel) stability of grid orientations. In our pre-registered hypotheses, we expected to detect both temporal and spatial stability, and that these measures would differ between age groups. Across all participants, temporal stability did not significantly exceed chance levels at 50% (t(109) = 1.18, p = 0.121, bf_01_ = 8.99), with a mean stability of 50.78%, suggesting high within-subject variability in grid orientations over time. We found no significant difference in temporal stability between younger and older adults with mean values of 50.14% (Older) vs. 51.02% (Younger).

**Fig. 3.**
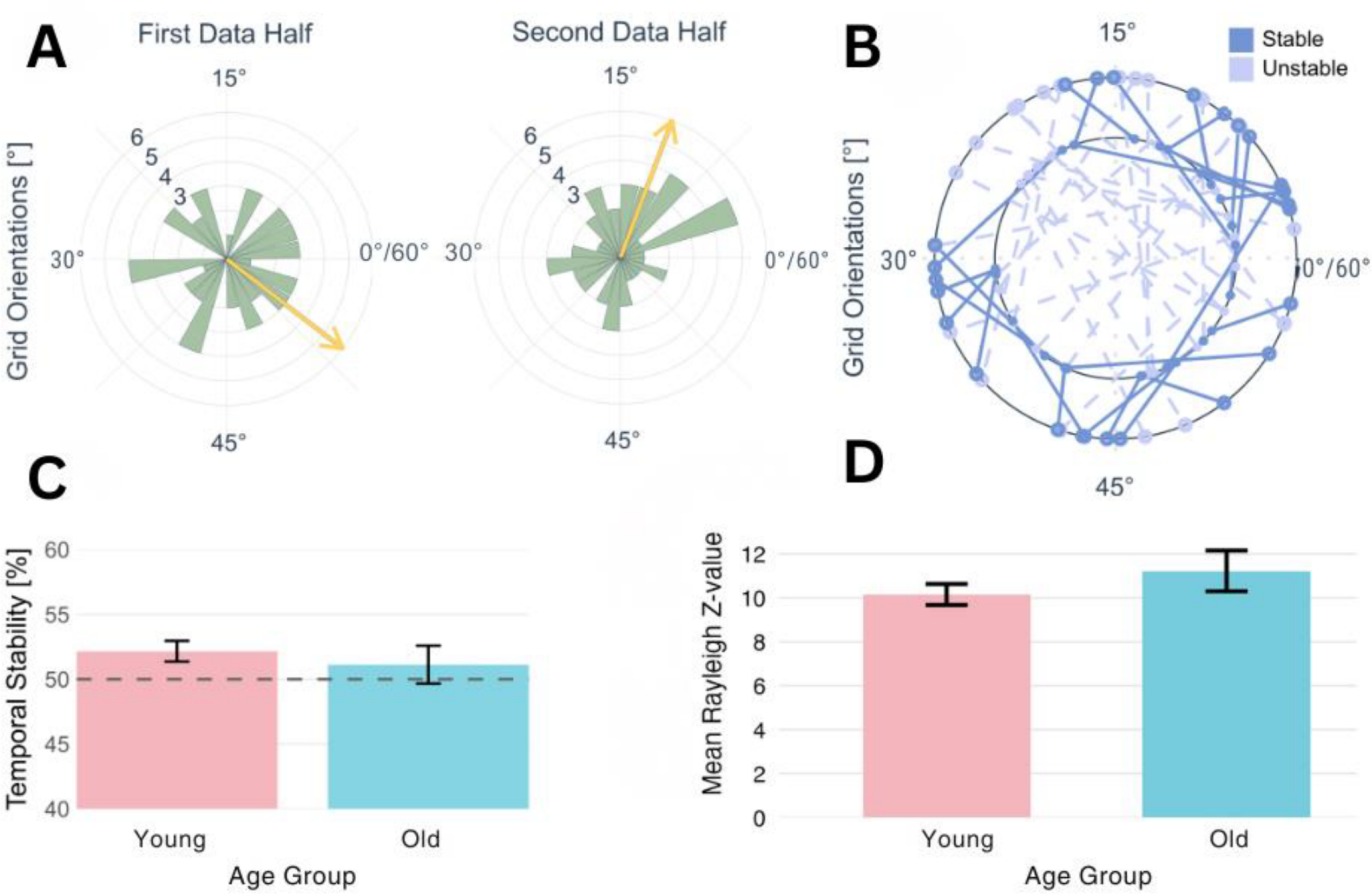
Temporal and spatial stability measures. (A) Circular histogram showing the distribution of preferred 6-fold orientations for voxels in an exemplary participant. The yellow arrow indicates the mean preferred direction across all voxels. The absence of a dominant orientation suggests a lack of spatial stability. (B) Voxel-wise temporal stability across two data halves for an exemplary participant. The inner circle represents the preferred orientation in the first half, while the outer circle represents the second half. Darker blue points indicate stable voxels (within 15° between halves), while lighter blue points represent unstable voxels. Each voxel’s orientation is connected across time with a solid or dotted line. (C) Proportion of temporally stable voxels in younger and older participants. Neither group showed a significant deviation from chance level (50%, dotted line), and there was no significant difference between groups. (D) Spatial stability of grid-like orientations across voxels, quantified using Rayleigh’s test for uniformity. Higher Z-values indicate stronger clustering of preferred orientations. No significant differences were observed between age groups. Error bars represent the standard error of the mean (SEM).

Spatial stability, assessed using Rayleigh’s test for non-uniformity of voxel orientations, revealed that 48.7% of younger participants and 51.7% of older participants showed significant spatial clustering—proportions that substantially exceed the 5% chance level (p < 0.001 for either group). Mean Rayleigh Z-values did not differ between younger (M = 10.2, SD = 4.3) and older participants (M = 11.2, SD = 5.0), t(44.0) = 1.03, p = 0.31, bf_01_ = 3.184 (see Fig. 3). These findings contrast with prior work showing reduced temporal stability in older participants when compared to a younger group, but no significant group differences in spatial stability (Stangl et al., 2018). In our analyses, temporal stability, a prerequisite for detecting GLS using fMRI, was no higher than chance in either group. While spatial stability was present in approximately half of our participants, this proportion is lower than the 81% reported by Doeller et al. (2010).

### 3.3 Control analyses: Data processing choices

Given that our pre-registered analyses did not confirm the expected GLS effects, we conducted additional analyses to examine whether methodological and cognitive factors influenced our findings. Spatial smoothing is commonly applied in fMRI preprocessing to improve signal-to-noise ratios but may also blur fine-grained neural representations, potentially affecting grid-code estimates. While our pre-registered analyses were conducted on unsmoothed data, we repeated them using spatially smoothed data (Gaussian kernel, 4 mm FWHM). The results showed a strong correlation between smoothed and unsmoothed GLS estimates (r = 0.887, 95% CI [0.838, 0.922], p < 0.05), indicating a modest impact on overall grid-code detection. However, smoothing introduced subtle differences: a significant 7-fold signal emerged in the right EC, rather than the left, for the full sample (t(109) = 1.87, p = 0.031, bf_01_ = 1.75), although it did not persist in age-stratified analyses. In contrast, the 6-fold age-group difference in the right EC remained significant, mirroring our initial findings.

Beyond smoothing, we also considered whether segmentation methods affected GLS detectability. Our primary analyses relied on a manually segmented subset of 110 participants. To validate these findings in the full sample and assess potential effects of the segmentation method, we reran analyses using automatically segmented masks across all 207 participants (see Table 2 for full sample demographics). Results remained largely consistent: with no detection of population level GLS effects (all p > 0.05), but with age-group effects persisting (younger participants, n = 137; older participants, n = 66; t = 1.963, p = 0.026, bf_10_ = 1.30, d = 0.29). A Pearson correlation between grid-code measures from manually and automatically segmented masks (r = 0.562, 95% CI [0.416, 0.679], df = 105, p < 0.05) indicated moderate agreement between methods.

Finally, recognising the inherent challenges in capturing EC signal quality, we assessed whether low tSNR could account for the absence of robust GLS effects. Signal quality in the EC is inherently challenging due to its anatomical location and susceptibility to signal dropout in fMRI. To address this limitation and ensure that the lack of signal was not due to poor signal quality, we compared tSNR across multiple steps in the preprocessing. We assessed tSNR at three stages, raw single-echo data (TE = 30.9 ms), optimally combined multi-echo data (TEs = 12.2 ms, 30.9 ms, 49.6 ms) and the final dataset processed with both a 24-parameter denoising strategy and multi-echo ICA-based denoising using Tedana. As expected tSNR increased progressively across these processing stages (see Fig. 1), with the highest signal stability observed in the fully processed data. There was no significant correlation between the tSNR and GLS magnitude in the final data set (r = -0.144, p = 0.134), and independent-samples t-tests confirmed that tSNR did not significantly differ between younger (M = 47.7, SD = 5.1) and older (M = 46.7, SD = 5.3) participants (t(50.7) = 0.91, p = 0.37, bf_01_ = 3.1). Mean framewise displacement (FD) differed significantly between age groups, with older participants showing higher motion than younger participants (Younger: M = 0.025, SD = 0.024; Older: M = 0.050, SD = 0.044; Welch’s t(35.6) = −2.97, p = 0.005, bf_10_ = 110.23). However, mean FD was not significantly associated with grid-like signal magnitude in either fMRI run (run 1: r = −0.10, p = 0.28; run 2: r = −0.17, p = 0.08), indicating that individual differences in head motion did not explain variability in grid-code estimates. These findings confirm that the multi-echo approach successfully improved signal stability, reducing the risk of signal dropout as a confounding factor. Taken together, these control analyses suggest that neither spatial smoothing, segmentation method, nor signal quality can fully account for the lack of robust population-level GLS effects.

### 3.4 Analyses of high-performance participants

In the second exploratory analysis, we investigated whether lower engagement led to lower GLS. For this, we used object-location memory scores as an indirect proxy for overall task engagement in the virtual environment. Participants exhibited strong ceiling effects with 88% of the younger group and 53% of the older group scoring 6 or above out of 7 possible target hits (see Fig. 1). Analyses revealed significant age-group differences in behavioral performance (t(38.33) = 4.44, p < 0.001, bf_10_ = 11809, d = 0.97). When excluding the lower scoring participants, with scores below 6, GLS remained non-significant in both age groups. Follow-up analyses showed no significant correlation between task scores and GLS magnitudes (r = 0.16, p = 0.10), though age negatively correlated with task performance (r = -0.53, p < 0.01).

### 3.5 Unimodal signal interference

The detection of a 7-fold modulation in the BOLD signal, observed in the left EC across the overall sample, and particularly within the older group, deviates from the canonical 6-fold symmetry typically associated with grid-cell firing patterns. Although Bayesian analyses characterised this signal as anecdotal, we nonetheless explored the potential sources for this non-canonical symmetry. Recent findings by Newton et al. (2024) suggest that non-6-fold modulations could result from a compensatory reliance on head direction cues. Specifically, a strong unidirectional (1-fold) signal—reflecting consistent activity in a specific movement direction—was linked to poorer path integration performance.

To determine whether a directional bias was present in our data, we tested the 1-fold signal magnitude against zero. One-sample t-tests revealed no significant 1-fold modulation in either the older (t = 1.47, p = 0.149) or younger (t = -0.59, p = 0.560) groups. We then examined the relationship between the 1-fold signal and behavioural task scores. Similar to our 6-fold analysis, no significant correlation was found. We then conducted a linear mixed-effects model to assess whether age, hemisphere, or sex influenced the 1-fold signal. None of these factors showed a significant effect on 1-fold signal (all main and interaction effects: p > 0.188). To further investigate the presence of unidirectional bias, we employed LMMs to assess whether the strength of the 1-fold signal predicted changes in either the 6-fold or 7-fold grid code signals. Two separate models were run: one examining whether stronger 1-fold signals predicted increased 7-fold signal strength, and another testing whether stronger 1-fold signals predicted weaker 6-fold signals, both models including interactions with hemisphere and continuous age. No significant relationships were found between the 1-fold signal and either the 7-fold and 6-fold signals, nor were any significant interactions with hemisphere or age detected.

## 4 Discussion

Our pre-registered hypothesis proposed that entorhinal grid-cell-like representations would be negatively related to age, predicting that younger participants would exhibit stronger GLS compared to older participants. While we did observe some age-related differences in grid magnitudes, these findings were inconsistent and did not support our pre-registered expectation in all analyses. Specifically, age-group comparisons showed significant differences in accordance with prior work (Stangl et al., 2018), but GLS were not significantly different from zero, even when examining just the younger group. Notably, the right EC in younger participants, the region and group most consistently associated with GLS in prior work (Doeller et al., 2010; Stangl et al., 2018), showed our strongest 6-fold effect, but it only approached significance (p = 0.068). At the same time, an unexpected 7-fold modulation in the left EC (p = 0.009) exceeded our strongest 6-fold result, despite control analyses being intended to rule out non-hexagonal periodicities. Together, this pattern suggests that the small deviations from zero we observed are more compatible with weak or non-specific directional modulation than with a robust, grid-specific hexadirectional signal in this passive paradigm. Consistent with this interpretation, Bayesian follow-up analyses indicated that the evidence for both the age-group difference and the 7-fold effect was inconclusive.

Given the inconsistencies in our findings, we conducted additional exploratory analyses to determine whether analytical choices contributed to the absence of consistent GLS effects. A potential concern is whether spatial smoothing affects GLS detectability. Smoothing is commonly applied in fMRI preprocessing to improve signal-to-noise ratios, but it may also reduce the specificity of localised neural signals, such as grid-like representations. While our initial analyses used unsmoothed data, we repeated our pre-registered analyses using spatially smoothed data. The resulting GLS estimates were highly correlated between smoothed and unsmoothed data. Group differences in GLS persisted, whereas GLS expression at the population level remained non-significant.

Another methodological factor that could influence results is EC segmentation. The EC is anatomically complex, with high inter-individual variability and few clear structural boundaries, making both manual and automatic segmentation challenging (Price et al., 2010). To assess whether segmentation approach influenced our findings, we reanalysed our data using automatically segmented EC masks instead of manual segmentations. While automatic segmentation is faster and less labour-demanding, it may come at the cost of reduced anatomical specificity, likely incorporating portions of the perirhinal cortex, which could dilute true entorhinal signals. Despite this, our findings remained unchanged—GLS were not significantly different from zero, but age-group differences persisted.

To rule out the possibility that dropout or low tSNR contributed to the absence of detectable GLS, we compared tSNR at different data processing steps, which showed that our multi-echo fMRI approach substantially boosted tSNR and mitigated potential signal dropout in the EC. Despite these improvements, we observed no significant correlation between tSNR and GLS magnitude, indicating that signal quality alone is unlikely to explain the absence of detectable GLS. Moreover, these findings align with recent work by Newton et al. (2024), who used high-resolution imaging (1 mm slices at 7 Tesla) to reduce through-plane susceptibility distortions, a common cause of signal loss in regions like the EC. Despite their advanced method, they similarly failed to detect GLS under a passive navigation paradigm. This convergence of null results across studies suggests that neither dropout nor low tSNR can account for the absence of detectable GLS in our dataset.

We further investigated a potential disruptive one-fold signal as a potential confound. A one-fold signal has been implicated in compensatory reliance on head direction cues when grid-like representations are weak. Newton et al. (2024) found that participants who performed worse on path integration tasks exhibited a stronger 1-fold signal, suggesting that head direction signals may dominate when grid-like representations are insufficient. Beyond the absence of robust GLS, we also observed a 7-fold periodicity in the EC—an effect that deviates from the well-established 6-fold symmetry of grid-cell activity, raising questions about its origin. One possibility is that the 7-fold signal reflects a disruption of the expected 6-fold symmetry due to a competing unidirectional signal. However, our analyses did not reveal strong 1-fold effects, suggesting that reliance on head direction cues was not the primary driver of this pattern. Notably, the significant 7-fold effect shifted from the left EC in our primary (unsmoothed) analysis to the right EC when spatial smoothing was applied. This sensitivity to preprocessing further undermines the likelihood of it representing a stable, biologically meaningful neural phenomenon and reinforces the possibility that the 7-fold signal reflects spurious noise.

A notable limitation of our paradigm is the relatively short task duration and limited sampling of trials per movement direction. We acquired approximately 20 minutes of fMRI task data, whereas several previous GLS studies have used substantially longer scanning durations, in some cases exceeding 1 hour (Kunz et al., 2015; Stangl et al., 2018). Recent methodological work suggests that designs with many participants and shorter sessions can often maximize statistical power for simple contrasts (Miller, 2024), and that sample size and scan time may be partially interchangeable for certain prediction tasks (Ooi et al., 2025). However, grid code analysis presents unique challenges, as accurate estimation of voxel-wise grid orientations across 36 directional bins requires sufficient sampling per direction. Our study’s shorter duration contributed to obtaining only 3–4 trials per movement direction per run, resulting in sparse data for standard split-half analyses that likely compromised the reliable estimation of grid orientations. While longer scan durations could increase sampling density, Newton et al. (2024) employed 30 minutes of task time and also reported null findings, and extending scan duration in passive paradigms may come at the cost of reduced attention and increased fatigue.

Beyond data quantity, the spatial resolution of the fMRI acquisition (2.6 mm isotropic) limits interpretation. This voxel size can cause partial volume effects at the entorhinal cortex boundaries, potentially diluting region-specific signals. This resolution also averages neural activity across large voxel volumes, meaning grid orientations are estimated from a wider range of cells with potentially more varied grid orientations. Yet, given that spatial smoothing, which similarly averages signals, is frequently employed in studies that successfully detect GLS, resolution limitations may not be the sole explanation for our results. Additionally, our analyses treated the entorhinal cortex as a single region of interest. However, grid cells in rodents are localized to the medial entorhinal cortex, and human homologs of this subregion have been identified in posterior-medial EC (Maass et al., 2015; Navarro Schröder et al., 2015). Recent work suggests even more complex organization, with three band-like functional zones traversing the EC (Reznik et al., 2024). Our whole-EC approach may therefore have diluted signals localized to specific sub-regions.

A key similarity between the present study and the recent work by Newton et al. (2024) is the use of passive navigation paradigms. Unlike active, real-world navigation, which integrates multiple self-motion cues, including proprioceptive, vestibular, and visual signals such as optic flow and motion parallax, fMRI navigation paradigms inherently limit the availability of these body based inputs, leaving visual cues as the primary driver of perceived self-motion. Yet, many active fMRI studies successfully detect GLS using similar visual information and also lacking full body cues. The key difference likely lies not in the visual cues themselves, but in the absence of crucial factors linked to active control, such as motor planning, internal motion prediction, or heightened cognitive engagement in passive paradigms. This aligns with rodent studies where passive transport, despite available visual/vestibular cues, disrupts grid cell firing (Winter et al., 2015), suggesting some active processing is fundamental for maintaining the grid code. Human neuroimaging has long shown dissociable network engagement across navigation modes, with active wayfinding selectively engaging hippocampus and route following engaging caudate (Hartley et al., 2003), indicating a dissociation by navigational mode: agency, goals, and sampling strategy rather than visual input alone.

The importance of internally driven processes is highlighted by evidence that GLS emerge even during purely imagined navigation (Bellmund et al., 2016) comparable to GLS in active virtual navigation (Horner et al., 2016). Yet, the role of visual input is complex, particularly considering potential species differences where primates may rely more heavily on visual or view-based spatial representations compared to rodents (Martinez-Trujillo, 2025). Consistent with this, primate electrophysiology shows that active visual exploration alone can engage the grid system. Killian et al. (2012) found grid-like firing in monkey entorhinal cortex linked to gaze position during free viewing of static images, without locomotion. By contrast, in freely moving macaques, grid-modulated cells are rare. Mao et al. (2021) report a rare ≈2% grid fraction in hippocampal–entorhinal recordings, with activity dominated by head-facing location, allocentric direction, and strong eye-movement modulation rather than canonical grid periodicity. Across species, GLS expression appears conditional on both movement mode and dominant inputs: rodents show strong locomotion-anchored grid codes, whereas primates also show view and eye-movement-related modulation. If free viewing is enough to elicit GLS in primates, why do we not detect them in passive fMRI? One possibility is that the active, self-directed visual sampling via eye movements is fundamentally different from the passive viewing in our paradigm, where there is less need for active visual search. Alternatively, the necessary inputs for eliciting grid like responses differ across species as human MTL networks may only be minimally coupled to unimodal sensory systems, whereas the MTL of non-human primates receive direct inputs from high-order unimodal association cortices (Reznik et al., 2023). Ultimately, these complexities highlight the need for caution in interpreting human fMRI GLS data, acknowledging that macroscopic signals in humans may not perfectly mirror the cellular mechanisms detailed in rodent, or even non-human primate models due to both species-specific adaptations and methodological differences.

A further potential explanation for the null findings may be differences in task-engagement. Passive viewing paradigms place significantly lower cognitive demands on participants, potentially resulting in reduced attentional engagement or insufficient neural synchronization necessary for robust GLS. While we attempted to explore this possibility through the object-location memory task as an indirect measure of task-engagement, our analyses revealed no significant association between task accuracy and GLS magnitude. However, ceiling effects in the behavioural task, especially in younger participants, may have reduced our sensitivity to any link between task-engagement and GLS. While good object-location memory necessarily implies that participants attended to and encoded object positions to some extent, this measure is not a direct assessment of attention and offers only a coarse index of engagement. Because performance was high for most participants, the task may have been insufficiently demanding to capture meaningful variability in engagement.

Inconsistencies in GLS detection are not unique to passive paradigms, however. The signal strength of GLS is susceptible to individual differences, as demonstrated by the age-related (Stangl et al., 2018) and genetic risk effects (Kunz et al., 2015), as well as by factors of task design like movement speed (Doeller et al., 2010). Beyond its mere amplitude, the form of the signal has also been shown to change. For example, a 4-fold periodicity, rather than the canonical 6-fold, is observed in early blind participants (Sigismondi et al., 2024), and can also be induced by environmental barriers (He & Brown, 2019). In the same vein, Wagner et al. (2023) reported a clear six-fold signal while participants observed another agent’s movement, but no six-fold signal during self-navigation; instead, a 4-fold modulation emerged in the active phase. Collectively, these findings suggest that grid-like signals are highly context-dependent and highlight the critical need for systematic investigation into the precise conditions that govern their expression and stability.

Despite the unexpected findings, our pre-registered study offers key strengths that contribute to the broader discussion on GLS in human fMRI. Our findings, particularly when considered alongside recent work by Newton et al. (2024), raise important questions about the conditions under which GLS can be detected, and warrant considerations of how passive viewing paradigms are used. Moving forward, directly comparing active and passive navigation paradigms, while controlling for and dissociating the influence of attention will be crucial. Furthermore, systematically assessing the impact of study parameters, such as passive or active navigation, task durations, directional bin sampling or EC segmentation strategies, is essential to define requirements for robust GLS detection and clarify the factors governing stable grid-like representations at the macroscopic level.

## Supplementary Material

Supplementary Material

## Data Availability

The study pre-registration can be found at https://aspredicted.org/rfx5-gmmq.pdf. The code used for the analyses is available at https://github.com/jokran/GLS_fMRI_Analysis_Kransberg_2025. The LCBC dataset has restricted access, but requests can be made to the corresponding author, and some of the data can be made available given appropriate ethical and data protection approvals.
