# Supplementary material for: Failure to detect entorhinal grid-like signals in a passive navigation human fMRI study
Source: Imaging Neurosci (Camb). 2026 Apr 7;4:IMAG.a.1196. doi: 10.1162/IMAG.a.1196 (PMC13058850; doi:10.1162/IMAG.a.1196)
Supplement: Supplementary Material [file IMAG.a.1196_supp.pdf]

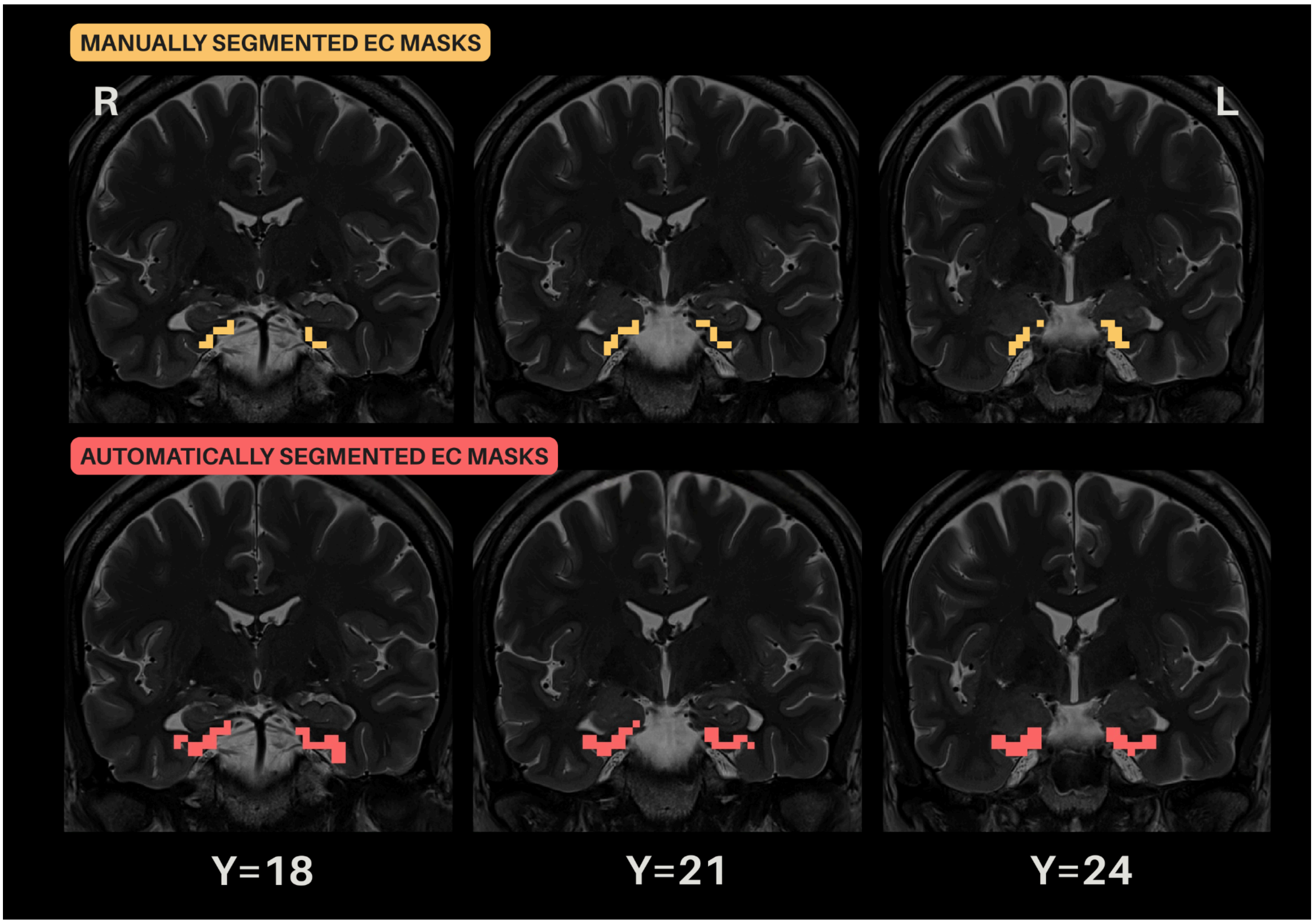

Supplementary Figure 1. Coronal slices illustrating manually (top, yellow) and automatically (bottom, red) segmented entorhinal cortex (EC) masks in an exemplary younger participant. Slice positions are given as Y-coordinates along the anterior–posterior axis.. **L** and **R** denote left and right hemispheres. Manual segmentations followed the protocol of Berron et al. (2017), and automatic EC masks were derived using FreeSurfer (v7.3.2).
